# Esophageal Liposarcoma: A Case Report and Review of the Literature

**DOI:** 10.7759/cureus.48759

**Published:** 2023-11-13

**Authors:** Faizan Boghani, Evan C Compton, Gregory Postma, Amanda Barrett

**Affiliations:** 1 Pathology, Augusta University Medical College of Georgia, Augusta, GEO; 2 Otolaryngology, Augusta University Medical College of Georgia, Augusta, GEO

**Keywords:** mesenchymal tumor, esophageal cancer, giant esophageal polyp, esophageal neoplasms, esophageal liposarcoma, esophagus, liposarcoma

## Abstract

Liposarcomas are extremely rare occurrences in the esophagus. Here, we present an unusual case of esophageal liposarcoma that contributed to a long history of dysphagia before a definitive diagnosis was reached. The case is that of a 61-year-old woman who complained of dysphagia and foreign body sensation in her throat and was found to have a large filling defect within the cervical esophagus on barium esophagogram. She underwent endoscopic resection of the mass and was diagnosed with a five-centimeter long, well-differentiated esophageal liposarcoma, confirmed with fluorescence in situ hybridization for *MDM2* gene locus amplification. Subsequent laser ablation of the tumor bed was conducted with no recurrences noted to date. Proper histologic identification, alongside adjunctive cytogenetic and molecular diagnostics, followed by definitive surgical resection and extended follow-up, are emphasized as critical in optimizing outcomes for this disease. A review of the existing English-language medical literature relating to esophageal liposarcoma was performed and summarized.

## Introduction

Liposarcomas are the most common malignant soft-tissue tumor in adults, representing 15%-25% of all sarcomas, and they most commonly arise in the extremities and retroperitoneum [1]. They are exceedingly rarely found in the gastrointestinal (GI) tract, and even more so in the esophagus where they comprise 1.2%-1.5% of all GI liposarcomas [2]. They are derived from primitive mesenchymal cells and usually arise from the esophageal submucosa [3]. Since the publication of the first case report in 1983, 76 cases of esophageal liposarcoma have been reported in the English-language medical literature [2-16]. The clinical presentation of these tumors is usually with symptoms of dysphagia, dyspnea, or, in some cases, regurgitation of the mass [2]. Evaluation often reveals a pedunculated intraluminal mass, which may be visible on contrast esophagography [3,4]. Esophageal liposarcomas are associated with a better prognosis than their conventional counterparts at other sites and usually do not metastasize from their primary location [5]. The WHO histological classification includes well-differentiated, dedifferentiated, myxoid, and pleomorphic subtypes [1]. Imaging studies, such as magnetic resonance imaging (MRI), computed tomography (CT), and positron emission tomography (PET), may be useful in the diagnostic process and surgical planning [4,6]. Biopsy and immunohistochemistry for *MDM2* gene locus amplification remain critical for diagnosis [7]. The primary treatment for esophageal liposarcoma is surgical; the choice of open esophagotomy versus endoscopic approach depends upon individual tumor characteristics [3]. Local recurrence has been described in up to 10% of cases, emphasizing the importance of complete resection with negative margins and long-term follow-up [8]. The aim of this report is to describe a case of giant well-differentiated esophageal liposarcoma that was successfully resected with an endoscopic approach with no recurrence to date at 12 months and to conduct a review of the existing English-language medical literature relating to esophageal liposarcoma.

## Case presentation

A 61-year-old woman was referred for longstanding dysphagia. Thirteen years prior, she was evaluated for dysphonia and diagnosed with laryngopharyngeal reflux and muscle tension dysphonia. Notably, she described a sensation of a "big flap that comes up my throat," "gag[s] me," and "block[s] my airway.” She also reported difficulty swallowing solids, liquids, and especially pills. She had multiple esophagogastroduodenoscopies (EGDs) with empiric dilation eight years ago, but no diagnosis prior to being evaluated at our center.

At the time of the current presentation, the patient denied any other symptoms, including fever, chills, weight loss, or hemoptysis. Physical exam was unremarkable with the exception of slight dysphonia, consistent with the patient’s prior history. Head and neck exam was normal, including upper airway evaluation with nasopharyngoscopy.

**Figure 1:**
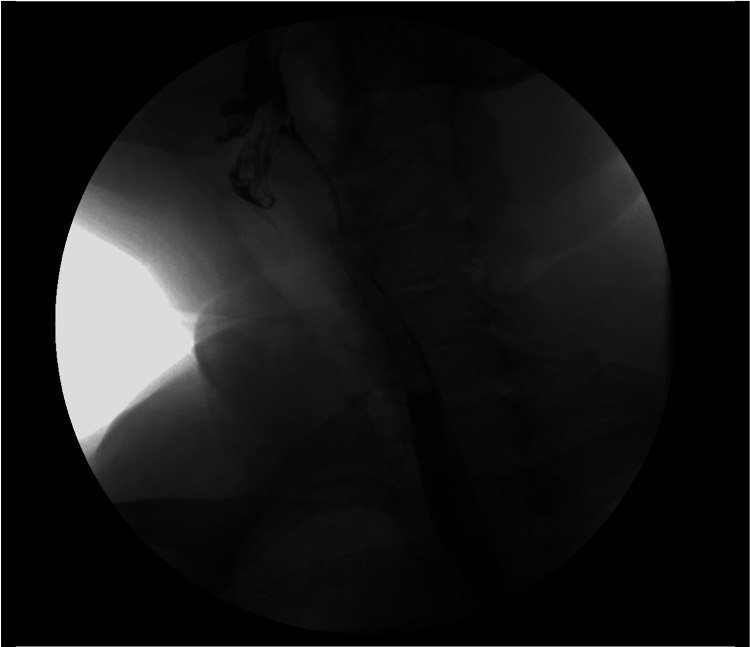
Barium esophagogram demonstrating an intraluminal mass in the cervical esophagus

A barium esophagogram was obtained for further evaluation. A long smooth tubular filling defect within the proximal esophagus, approximately 9 cm in length, was observed beginning at the level of the cricopharyngeus muscle (Figure 1). This was interpreted on radiology report to possibly represent a pedunculated lipoma or mural associated mass in the posterior esophagus. The findings were discussed with the patient and a plan was made to pursue endoscopic excision in the OR.

The patient underwent rigid esophagoscopy with a Slimline scope to expose the mass. A zero-degree telescope was used to visualize the mass from the left hypopharyngeal wall, and suction cups were used to pull the elongated mass from the esophagus. Next, an approximately 6 cm mass was truncated using cupped forceps and Sonicision (Covidien, Ireland) at the left hypopharyngeal wall and retrieved transorally without mucosal tear. The mass was sent for permanent section to pathology. The patient tolerated the procedure well with no complications and was discharged from the hospital on the same day.

**Figure 2:**
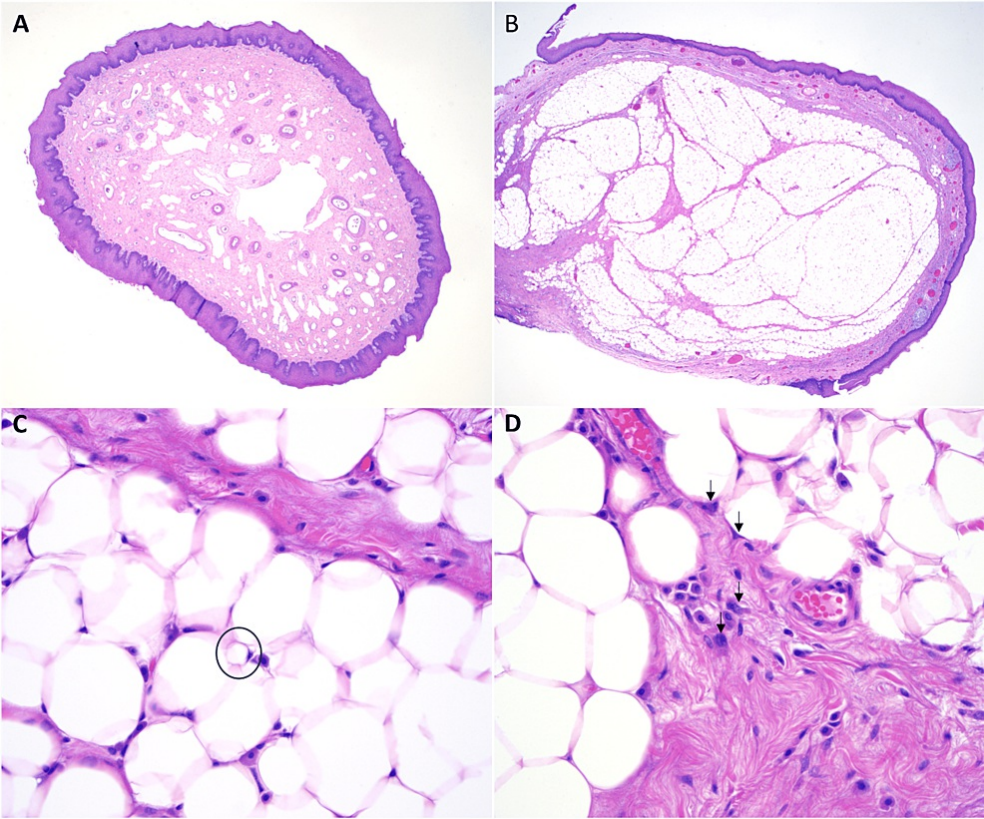
Hematoxylin and eosin (H&E) stained sections of liposarcoma. Low-power (20x) examination of the mass showed cross sections with a predominant fibrovascular core (A) and more distal sections showing more abundant adipose tissues (B). Higher power examination of the adipose tissue (400X) showed scattered lipoblasts (C, circled), with hyperchromatic nuclei indented by multiple lipid vacuoles. Examination of the fibrous areas also showed scattered, hyperchromatic, and atypical nuclei (D, arrows).

**Figure 3:**
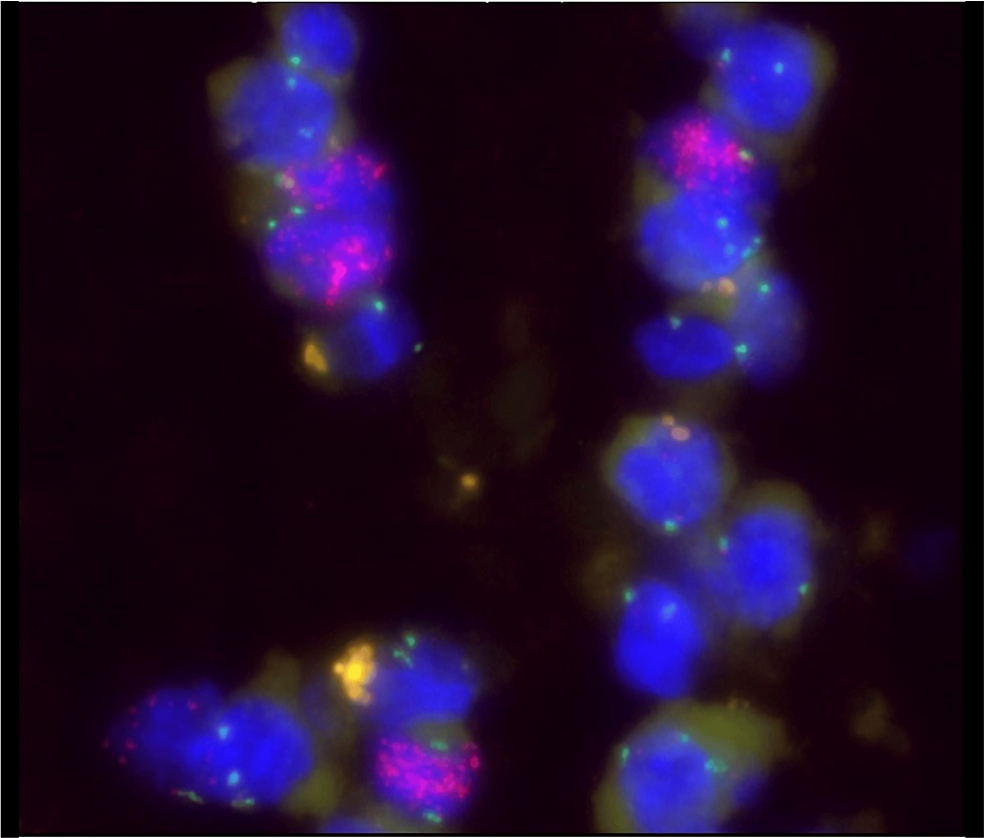
MDM2/CEN12 dual-color fluorescence in situ hybridization. A ratio of 7.0 was observed in the nuclei, demonstrating amplification of the MDM2 locus.

Pathology described the specimen as a single 5.0 cm × 0.7 cm × 0.4 cm long, tubular, fleshy pink-tan mass. Histologically, the polypoid mass was noted to be surfaced by normal squamous mucosa, with a central core of adipose tissue at the pedunculated end, which contained occasional scattered hyperchromatic, enlarged, atypical nuclei with irregular contours (Figure 2). Fluorescence *in situ* hybridization (FISH) analysis using a dual-color *MDM2/CEN12* probe set was performed to detect *MDM2* gene amplification and was positive (Figure 3), supporting the final diagnosis of a pedunculated Grade 1 well-differentiated esophageal liposarcoma. The final resection margin at the stalk portion of the mass was negative for definitive involvement based on hematoxylin and eosin (H&E) evaluation.

The patient’s case was discussed at the Multidisciplinary Head & Neck Tumor Board. All available historical data, radiology, pathology, and physical exam findings were reviewed in detail with the group. A recommendation was made that for this patient’s T1aNxMx liposarcoma of the upper esophagus, she should undergo direct laryngoscopy and esophagoscopy with laser ablation and re-excision of margins to ensure clearance of disease, with subsequent close observation. The tumor board’s recommendation was discussed with the patient, and she was amenable to re-excision.

The patient underwent esophagoscopy to further examine the upper esophageal sphincter and site of prior tumor. There was no evidence of residual tumor noted on examination. There was a left-sided esophageal web at the upper esophageal sphincter (UES). CO_2_ laser was then used under microscopy to ablate the mucosal bed where the tumor was previously excised.

A CT scan of the neck and chest with contrast was obtained following the operation and revealed no residual disease or evidence of local, regional, or distant metastases.

On follow-up visit one month after re-excision, the patient reported complete resolution of dysphagia and dysphonia. Six months after re-excision, the patient reported intermittent solid food dysphagia. The patient underwent repeat esophagoscopy in the OR, and there was no tumor noted on examination. A small left-sided esophageal web was again visualized, and this area was successfully dilated to 84 Fr with a balloon.

The patient denied dysphagia during follow-up visits at nine and 12 months, respectively, following balloon dilation of the small esophageal web. Head and neck exam at each of these visits was normal, including upper airway evaluation with nasopharyngoscopy. She continues to do well and has been instructed to follow up on an as-needed basis.

## Discussion

Liposarcomas are a class of malignant tumor derived from adipose tissues [1]. They are the most common malignant mesenchymal tumor in adults, comprising 15%-25% of adult sarcomas [1]. Liposarcomas are a group of highly diverse tumors with highly heterogeneous histological and clinical characteristics, which emphasize the need for further sub-classification. Per WHO classification, they can be further divided into well-differentiated, dedifferentiated, myxoid, and pleomorphic subtypes [1]. Well-differentiated liposarcoma, the tumor presented in this patient’s case, is the most common subtype and is characterized cytogenetically by giant marker and ring chromosomes and by the amplification of the *MDM2* gene locus, an inhibitor of p53, which can be identified either by immunohistochemistry or FISH as a diagnostic test [6]. The use of this test has been demonstrated in the literature to be of particular importance in avoiding misdiagnosis of an esophageal mass as a so-called benign giant fibrovascular esophageal polyp rather than a liposarcoma [7]. This is especially relevant as, unlike benign etiologies of an esophageal mass, liposarcomas can recur and efforts should be made during resection to ensure negative tumor margins with long-term surveillance following resection to screen for local recurrence.

Liposarcomas commonly arise in the sixth to seventh decades of life and usually involve the retroperitoneum or extremities [1]. Among well-differentiated liposarcomas at these typical sites, men and women are typically equally affected with the possible exception of the groin where a possible predilection for men has been reported [1]. They much more rarely involve the GI tract, with a reported incidence at autopsy of between 0.1% and 5.8% of liposarcomas [3]. The esophagus is an even rarer site, being involved in only 1.2%-1.5% of GI liposarcomas [2]. A review was undertaken to identify the total number of cases of esophageal liposarcoma reported in the English-language medical literature to date.

A search in PubMed was performed using the following search string: (oesophagus or esophagus or oesophageal or esophageal liposarcoma) and (Liposarcoma) or (“Esophageal Neoplasms”[MeSH], “Esophageal Diseases”[MeSH] and “Liposarcoma”[MeSH]) or (esophagus; liposarcoma[MeSH Terms]). All English-language articles and articles from 1983 (first case report) to May 2023 were included. A total of 76 cases of esophageal liposarcoma were identified in the English-language medical literature: 66 from two reviews [5,8] and 11 additional case reports from the review of PubMed as described above, with duplicates excluded [2-4,6,9-15]. Our case represents the 77th reported case of esophageal liposarcoma. The characteristics of these cases are summarized in Table 1.

**Table 1:** Demographic, clinical, and pathological characteristics of patients with esophageal liposarcoma

n=77		
Age, year	Median (range)	65 (38-84)
Sex	Male, n (%)	54 (70.1)
	Female, n (%)	23 (29.9)
Location of the tumor		
	Cervical esophagus, n (%)	49 (63.6)
	Upper esophagus, n (%)	12 (15.6)
	Middle esophagus, n (%)	1 (1.3)
	Lower esophagus, n (%)	8 (10.4)
	Thoracic, n (%)	2 (2.6)
	Not reported, n (%)	5 (6.5)
Site of the tumor		
	Intraluminal, n (%)	63 (81.8)
	Intramural, n (%)	14 (18.2)
Histology		
	Well-differentiated, n (%)	43 (55.8)
	Well-differentiated rhabdomyomatous, n (%)	2 (2.6)
	Myxoid, n (%)	9 (11.7)
	Pleomorphic, n (%)	1 (1.3)
	Dedifferentiated, n (%)	13 (16.9)
	Not specified, n (%)	9 (11.7)
Treatment		
	Endoscopic resection, n (%)	13 (16.9)
	Esophagectomy, n (%)	13 (16.9)
	Esophagotomy, n (%)	18 (23.4)
	Thoracoscopic resection, n (%)	16 (20.8)
	Gastrotomy, n (%)	1 (1.3)
	Not mentioned, n (%)	16 (20.8)
Recurrence, n (%)		9 (11.7)

Given the rarity of esophageal liposarcomas, no guidelines exist on the optimal management of this tumor. In this case, given the favorable size and location of the tumor, endoscopic resection was pursued and was successful. In other cases reviewed in the literature, more invasive surgical approaches have also been utilized, usually secondary to unfavorable tumor characteristics, such as size, location, or an intramural tumor, as opposed to an intraluminal pedunculated mass that is readily excisable at the level of a stalk. In such cases, the added morbidity associated with a more invasive approach, as well as the increased length of the recovery period and cost, may be justifiable in the pursuit of a complete resection. However, in cases similar to the one presented here, serious consideration should be given to an endoscopic approach to minimize the aforementioned factors while still achieving excellent outcomes.

The probability of recurrence is difficult to establish in esophageal liposarcoma, given the relative lack of data in the literature, which is almost exclusively comprised of single case reports with limited long-term follow-up, as well as the heterogeneity in clinical behavior of different subtypes of liposarcoma. Given that esophageal liposarcoma has been known to recur as late as 25 years post-initial resection, the need for long-term follow-up is emphasized [12]. The prognosis of esophageal liposarcoma remains excellent with adequate resection compared to liposarcoma of other sites given the decreased tendency of these tumors to metastasize [5].

## Conclusions

Esophageal liposarcoma are an exceedingly rare clinical entity that nevertheless should be considered in the evaluation of an esophageal mass. Definitive diagnosis requires histology, and the diagnosis of the well-differentiated subtype is greatly aided by the identification of *MDM2* gene amplification by immunohistochemistry or FISH. Resection is indicated and can be pursued either by invasive surgical approaches or alternatively by endoscopy when tumor morphologic characteristics are favorable. They can recur following resection, underscoring the need for negative tumor margins and extended follow-up.
